# Min–Max Optimal Control of Robot Manipulators Affected by Sensor Faults

**DOI:** 10.3390/s23041952

**Published:** 2023-02-09

**Authors:** Vladimir Milić, Josip Kasać, Marin Lukas

**Affiliations:** Faculty of Mechanical Engineering and Naval Architecture, University of Zagreb, HR-10000 Zagreb, Croatia

**Keywords:** robot manipulators, sensor faults, ℒ_2_-gain, min–max optimization, Newton-like algorithm

## Abstract

This paper is concerned with the control law synthesis for robot manipulators, which guarantees that the effect of the sensor faults is kept under a permissible level, and ensures the stability of the closed-loop system. Based on Lyapunov’s stability analysis, the conditions that enable the application of the simple bisection method in the optimization procedure were derived. The control law, with certain properties that make the construction of the Lyapunov function much easier—and, thus, the determination of stability conditions—was considered. Furthermore, the optimization problem was formulated as a class of problem in which minimization and maximization of the same performance criterion were simultaneously carried out. The algorithm proposed to solve the related zero-sum differential game was based on Newton’s method with recursive matrix relations, in which the first- and second-order derivatives of the objective function are calculated using hyper-dual numbers. The results of this paper were evaluated in simulation on a robot manipulator with three degrees of freedom.

## 1. Introduction

In recent years, the control of robot manipulators in the presence of faults, and also, in general, the control of nonlinear dynamic systems in the presence of faults, has been a very active area of research. The approaches have been primarily focused on systems affected by sensor faults [1,2,3], actuator faults [4,5] and simultaneously both sensor and actuator faults [6,7,8,9]. Many well-known advanced control methods have been proposed as a way to cope with fault occurrences: these methods include—but are not limited to—sliding mode control [10,11,12,13], adaptive control [14], model predictive control [15,16], artificial neural network control [17,18,19], fuzzy control [20], and hybrid control [21,22,23,24].

Furthermore, an optimal and robust control in the presence of sensor and actuator faults has, for the last decade, been a topic of import for researchers on the control of dynamical systems. Many authors consider the $L_{2}$-gain of a nonlinear system (often called nonlinear $H_{\infty }$, because, according to [25,26], the $L_{2}$-gain is equivalent to the $H_{\infty }$ norm of a linear system) to be a measure of the influence of the faults. A fixed $L_{2}$-gain fault-tolerant controller, for a class of Lipschitz nonlinear system with actuator saturation, was designed in [27]. In [28], a data-driven output-feedback approach to the fault-tolerant control (FTC) problem, considering $L_{2}$-gain properties, was proposed. A robust $H_{\infty }$ FTC scheme to regulate the quadrotor system was adopted in [29]: the approach was based on linear matrix inequalities (LMI), so the overall dynamics of the quadrotor were linearized. In [30], backstepping and adaptive control methods based on $L_{2}$-gain were combined together, to provide a passive fault-tolerant attitude controller of a nonlinear dynamic model of morphing aircraft.

All the above-mentioned works were based on the formulation of the problem in the form of LMI, or on the determination of the approximate solution of the associated Hamilton–Jacobi–Isaacs (HJI) equation. The application of LMI requires the linearization of system dynamics, and therefore robustness cannot be guaranteed in all operating points; on the other hand, solutions of the HJI equation can be complex, and therefore difficult to apply in real control tasks.

The difficulties described above motivated us to conduct the research presented here. We formulated the control problem of a robot manipulator affected by sensor faults, as a typical representative of nonlinear dynamic systems, in the form of optimal $L_{2}$-gain control and the related min–max optimization, and we approached its solution without LMI formalism or the need to approximate the solution of the HJI equation.

The main idea of the approach presented in this paper was based on the algorithmic procedure described in the authors’ previous works [31,32]. The idea of using a Newton-like algorithm with recursive matrix relations to calculate exact gradients and Hessians was applied to the synthesis of a control system which aimed to overcome the sensor malfunctions whilst maintaining desirable stability and optimal $L_{2}$-gain performance properties.

This paper considered the synthesis of the PID control law of a robot manipulator, which kept the influence of sensor faults below the permissible limits, and ensured the robust stability of the overall closed-loop system. The problem was presented as a two-player zero-sum differential game, with the objective function including a parameter in such a way that the control vector represented the “player” that minimized the objective function, while the vector containing the sensor faults represented the “player” that maximized the same objective function.

The algorithm proposed in this paper, in one part, was based on Lyapunov’s stability analysis. The derived stability conditions were used in the optimization process for the appropriate initial setting of the algorithm parameters: these conditions were crucial in the application of the bisection method by which we determined the minimum $L_{2}$-gain. Also, by using Lyapunov method, the stopping criteria of the algorithm was improved, by deriving inequalities dependent on PID controller gains that bound the distance of the current point from the saddle point, and thus added bound on the number of iterations.

The main contributions of this work are summarized as follows:

- A suitable mathematical tool and systematic algorithmic procedure for the synthesis of an optimal robust PID controller for robot manipulators affected by sensor faults was developed. The presented approach admitted a min–max formulation, and hence provided a guarantee of robustness, which was achieved by optimizing the worst-case performance. To the best of the authors’ knowledge, such an algorithmic PID controller synthesis procedure had not previously been investigated.
- In many other similar optimal control-based algorithms that have been proposed in the literature (see, for example, [33,34] and references therein), the nonlinear system dynamics were treated as equality constraints, and were included in the optimization process, using the method of Lagrange multipliers: the results were HJI equations that were very difficult or almost impossible to solve. For this reason, many approximation methods have been developed (see, for example, [35,36,37,38] and references therein) in which actual computational complexity increased with the number of system states which needed to be estimated. In contrast to such approaches, the algorithm proposed in this paper had no high-dimensional structure: this followed from the fact that, instead of incorporating the robot dynamics in the closed loop with the controller directly into an objective function, and solving the corresponding HJI equation, the state variables and PID controller gains were coupled by recursive matrix relations, used to calculate the first- and second-order derivatives that appear in the Newton-like method.

Based on the contributions noted above, the importance of the research presented in this paper lies in the fact that the proposed control architecture does not rely directly on sensor fault estimation, in order to improve the desired positioning of the robot manipulator, and is closely related to nonlinear $L_{2}$-gain robust control, where a PID-type controller, with its well-known simplicity of structure, is designed to be robust against a sensor fault in the system. Such a control strategy has the potential for practical applications, due to its simplicity of design, it having less lag between sensor fault appearance and accommodation, and it not requiring significant computing resources.

The rest of the paper is organized as follows. In Section 2, the robot manipulator dynamics and their main properties are presented. In Section 3, the min–max optimal control problem, of a robot manipulator affected by sensor faults and in a closed loop with a PID controller, is defined. The main results are presented in Section 4 and Section 5. Based on the theory of $L_{2}$ stability and Lyapunov’s approach, the stability conditions of the entire control system are established in Section 4. In Section 5, the bisection method, the Newton-like algorithm, the Adams discretization method and the recursive matrix calculation of gradients and Hessians are integrated into an efficient algorithmic procedure. The closed-loop response of the proposed control strategy and a robot manipulator with three degrees of freedom (3DOF) are evaluated in computer simulations, and the results are given and discussed in Section 6. Section 7 concludes the paper. There are three appendices included. Appendix A describes the notation used throughout the paper. In Appendix B, a detailed derivation of expressions for the kinetic and potential energy of the cylindrical robot manipulator is given. Appendix C contains the expressions for the evaluation of coefficients resulting from the mechanical properties of a cylindrical robot manipulator with 3DOF.

## 2. Dynamic Model and Properties

Consider a dynamic model of an *N*-DOF robot manipulator system of the form

(1) $$M\left(q\right)\;\ddot{q}+C\left(q,\dot{q}\right)\;\dot{q}+g\left(q\right)=u,$$

where $q\in R^{N}$ is the vector of generalized coordinates, $u\in R^{N}$ is the vector of control forces/torques applied to the system, $M\left(q\right)\in R^{N\times N}$ is the inertia matrix, $C\left(q,\dot{q}\right)\dot{q}\in R^{N}$ is the vector of centrifugal and Coriolis forces/torques and $g\left(q\right)\in R^{N}$ is the vector of gravitational forces/torques. It is well-known that Equation (1) can be obtained through modeling a robot manipulator system by Euler–Lagrange equations.

For this paper, we considered a robot manipulator that had both rotational and translational generalized coordinates, and hence had the following properties (see, for example, [39,40,41,42,43]):

**Property** **1.** *The matrix*

(2) $$\dot{M}\left(q\right)-2C\left(q,\dot{q}\right),$$

*is skew-symmetric for all $q,\;\dot{q}\in R^{N}$: this implies that*

(3) $$\dot{M}\left(q\right)=C\left(q,\dot{q}\right)+C{\left(q,\dot{q}\right)}^{T};$$

**Property** **2.** *The inertia matrix M(q) is a positive-definite symmetric matrix which satisfies*

(4) $$a_{1}{∥\xi ∥}^{2}\leq \xi^{T}M\left(q\right)\xi \leq \left(a_{2}+c_{2}∥q∥+d_{2}{∥q∥}^{2}\right){∥\xi ∥}^{2},$$

*for all ξ and $q\in R^{N}$, where $a_{1},\;a_{2}>0$, and $c_{2},\;d_{2}\geq 0$.*

**Property** **3.** *There exist constants, $c_{1}$ and $d_{1}$, such that the Coriolis and centrifugal term, $C\left(q,\dot{q}\right)\;\dot{q}$, satisfies*

(5) $$∥C\left(q,\dot{q}\right)\;\dot{q}∥\leq \left(c_{1}+d_{1}∥q∥\right){∥\dot{q}∥}^{2},$$

*for all q and $\dot{q}\in R^{N}$, where $c_{1}>0$, $d_{1}\geq 0$.*

As is well-known, the vector of gravitational forces/torques is a gradient of the potential energy. The considered class of robot manipulator was such that its potential energy depended linearly on translational generalized coordinates, while rotational generalized coordinates appeared as a trigonometric function, with period 2π. Hence, the following property was imposed:

**Property** **4.** *There exist positive constants, $k_{g1}$ and $k_{g2}$, such that the Jacobian of the gravity vector satisfies*

(6) $$∥\frac{\partial g(q)}{\partial q}∥\leq k_{g1}+k_{g2}∥q∥,$$

*for all $q\in R^{N}$.*

**Remark** **1.** *If the system (1) has no translational generalized coordinates, then $c_{2},\;d_{2},\;d_{1},\;k_{g2}=0$. See, for example, [44,45,46,47], and references therein.*

## 3. Optimal Control Problem

As robot manipulators often work in extreme environments, their sensors are prone to faults during operation: for example, due to environmental noise, like a magnetic field. Faults in robot manipulator sensors often occur due to vibrations that cause disruptions in communication between the control unit and the sensor, or even a short circuit. Furthermore, intermittent sensor connection, bias in sensor measurement and sensor gain drop can also be presented as faults.

For this paper, we were interested in designing a decentralized system controller (1) that guaranteed the stability around a desired constant of generalized coordinate positions $q_{d}\in R^{N}$, while the influence of sensor faults was kept under a permissible level. In this context, we considered the least upper bound (supremum) of the ratio of the $L_{2}$-norm of the vector of the output signals to be controlled, and the $L_{2}$-norm of the vector representing sensor faults. It should be noted that considering the control problem in this way was actually equivalent to the $L_{2}$-gain (or nonlinear $H_{\infty }$) optimal control defined in [25,26].

In a sensor fault situation, the internal robot dynamic properties are not affected, but the controller of the robot manipulator (1) receives generalized coordinates that are not exact, and are given as follows [2,8,48,49]:

(7) $$\begin{array}{cc} \bar{q} & =q+F_{p}\;\varphi_{p}, \end{array}$$

(8) $$\begin{array}{cc} \dot{\bar{q}} & =\dot{q}+F_{v}\;\varphi_{v}, \end{array}$$

where $\varphi_{p}\in L_{2}\left(\left[t_{0},\;t_{f}\right],\;R^{S}\right)$ and $\varphi_{v}\in L_{2}\left(\left[t_{0},\;t_{f}\right],\;R^{S}\right)$ are the vector functions representing sensor faults, and $F_{p}$ and $F_{v}$ are the matrices with appropriate dimensions. The assumptions about the sensor fault terms were employed as follows:

**Assumption** **1.** *There exist positive-definite time-dependent functions $f_{p}$ and $f_{v}$, such that the sensor faults are bounded by*

(9) $$\begin{array}{cc} ∥\varphi_{p}∥ & \leq f_{p}, \end{array}$$

(10) $$\begin{array}{cc} ∥\varphi_{v}∥ & \leq f_{v}. \end{array}$$

**Remark** **2.** *In many other works—for example, in [2,7,8,50]—sensor fault bounds are considered as known positive real constants. In this work, we did not treat these bounds as known specific constants, but as unknown functions that needed to be determined in such a way that they maximized the objective function, so that the proposed control strategy might be available not only for robotic manipulators but also for a wide class of nonlinear dynamical systems.*

A PID-type control law was proposed, with gravity vector compensation in the following form:

(11) $$\begin{array}{cc} u & =-K_{P}\;\tilde{q}-K_{D}\;\dot{\bar{q}}-K_{I}\;\upsilon +g\left(q\right), \end{array}$$

(12) $$\begin{array}{cc} \dot{\upsilon } & =\tilde{q}, \end{array}$$

where $\tilde{q}=\bar{q}-q_{d}$ was a generalized coordinates errors vector, and $K_{P}$, $K_{D}$ and $K_{I}$ were N×N positive–definite diagonal matrices. Note that, as $K_{P}$, $K_{D}$ and $K_{I}$ were diagonal matrices, the proposed control law was formed in a fully decentralized fashion.

**Figure 1:**
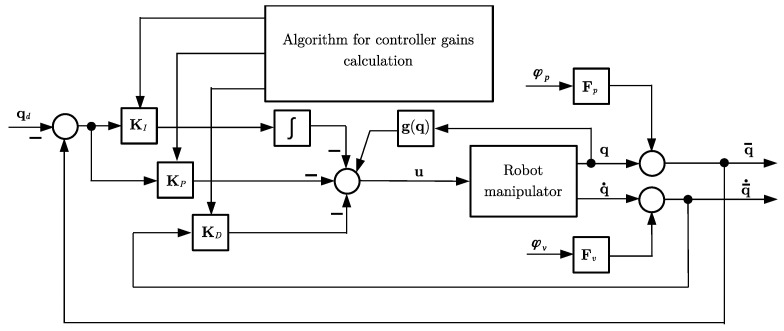
Block diagram of closed-loop system, with algorithm for controller gains calculation.

The robot manipulator system (1) with mixed rotational and translational generalized coordinates, affected by sensor faults (7) and (8) in a closed loop with a PID-type controller (11) and (12), can be represented by a block diagram, as shown in Figure 1. Each DOF of the robot manipulator was driven by servo motors with gears or pneumatic and hydraulic cylinders. The decentralized PID control law was implemented in the microcontroller, and point-to-point communication was established by electrical interface and physical layers. The received encoder data were used to close the feedback loop in the microcontroller.

Regarding the block diagram shown in Figure 1, in our approach the emphasis was not on the structure of the controller itself, but on the method for adjusting the gains of the controller. The PID controller structure was considered because it is the most common structure used in industrial applications.

**Remark** **3.** *It is important to emphasize the main difference between a conventional PID controller and our proposed one. In conventional applications of a PID controller, the gain values are independently and freely chosen by the designer, often using a trial-and-error method; however, in our approach, a proportional gain matrix $K_{P}$, a derivative gain matrix $K_{D}$ and an integral gain matrix $K_{I}$ were tuned automatically and correlatively by a numerical algorithm that will be presented in detail in the following sections.*

Inserting (7) and (8) into (11) and (12) gave

(13) $$\begin{array}{cc} u & =-K_{P}\;\hat{q}-K_{D}\;\dot{q}-K_{I}\;\upsilon -K_{P}\;F_{p}\;\varphi_{p}-K_{D}\;F_{v}\;\varphi_{v}+g\left(q\right), \end{array}$$

(14) $$\begin{array}{cc} \dot{\upsilon } & =\hat{q}+F_{p}\;\varphi_{p}, \end{array}$$

where $\hat{q}=q-q_{d}$.

Finally, inserting (13) and (14) into (1) gave closed-loop equations in the following form:

(15) $$\begin{array}{cc} M\left(q\right)\;\ddot{q}+C\left(q,\dot{q}\right)\;\dot{q} & =-K_{P}\;\hat{q}-K_{D}\;\dot{q}-K_{I}\;\upsilon -K_{P}\;F_{p}\;\varphi_{p}-K_{D}\;F_{v}\;\varphi_{v}, \end{array}$$

(16) $$\begin{array}{cc} \dot{\upsilon } & =\hat{q}+F_{p}\;\varphi_{p}. \end{array}$$

Based on the definition of finite $L_{2}$-gain and the definition of $L_{2}$ stability (see, for example, [25,51]), we defined the min–max optimal control problem of a robot manipulator affected by sensor faults, as follows:

**Problem** **1.** *Determine the PID controller gains, which are the elements of the matrices $K_{P}$, $K_{D}$ and $K_{I}$ in (11) and (12), and determine the “worst case” of sensor fault functions $\varphi_{p}$ and $\varphi_{v}$ in (7) and (8), such that the system (15) and (16) is stable around the desired constant generalized coordinates positions, the ratio of the $L_{2}$-norm of the vector of the output signals is controlled and the $L_{2}$-norm of the vector representing sensor faults is minimized. In other words, solve the following zero-sum differential game:*

(17) $$J_{\mu }^{*}\left(q_{0}\right)=\min_{K_{P},K_{D},K_{I}}\max_{\varphi }\;\left\{∥\hat{q}{∥}_{L_{2}}^{2}+{∥u∥}_{L_{2}}^{2}-\mu {∥\varphi ∥}_{L_{2}}^{2}\right\},$$

*where $\hat{q}$, $K_{P}$, $K_{D}$, $K_{I}$ and $\varphi ={\left[\varphi_{p}^{T}\;\;\varphi_{v}^{T}\right]}^{T}$ are coupled via closed-loop system dynamics (15) and (16), and μ>0 is a finite $L_{2}$-gain; furthermore, $q_{0}$ is an a priori known vector of initial states of generalized coordinates.*

## 4. $L_{2}$ Stability Conditions

It is well-known from the dissipativity theory [52,53,54] that a nonlinear dynamic system is $L_{2}$-stable if, for all initial conditions, all uncertainties w and all $t_{f}\geq t_{0}$ there exists a storage function *V*, which is also a Lyapunov function, such that the following inequality holds:

(18) $$\dot{V}\leq \gamma^{2}w^{T}w-z^{T}z,$$

where z is the performance variable, and γ>0 is the finite $L_{2}$-gain.

Note that the right-hand side of the inequality (18) in our case actually corresponded to the argument of the min–max operator in (17). Based on this observation, we give the following proposition, by which we set the stability conditions of the control system proposed in this paper:

**Proposition** **1.** *If the following conditions are satisfied:*

*(a)* 
(19) $$\alpha \lambda_{\min}\left\{K_{D}\right\}-\frac{1}{\alpha }\lambda_{\max}\left\{K_{I}\right\}-\alpha^{2}a_{1}>0,$$
*(b)* 
(20) $$\begin{array}{c} \alpha a_{1}+\left(c_{1}+d_{1}∥q∥\right)∥\hat{q}∥-\lambda_{\max}\left\{K_{D}\right\}-\mu \leq 0, \end{array}$$
(21) $$\begin{array}{c} \alpha \lambda_{\min}\left\{K_{I}\right\}-\alpha \mu \leq 0, \end{array}$$
(22) $$\begin{array}{c} 1-\lambda_{\max}\left\{K_{D}\right\}\sigma_{\max}\left\{F_{v}\right\}\leq 0, \end{array}$$
(23) $$\begin{array}{c} \alpha -\alpha \lambda_{\max}\left\{K_{D}\right\}\sigma_{\max}\left\{F_{v}\right\}\leq 0, \end{array}$$

*for some α>0, where μ>0 is a finite $L_{2}$-gain, and where $a_{1}$, $c_{1}$ and $d_{1}$ are defined in Properties 2 and 3, then the system (15) and (16) is locally $L_{2}$-stable.*

**Proof.**  The first step was to transform the system (15) and (16) into a form with a zero steady state. The stationary state of the system (15) and (16) is $\dot{q}=0$, $\hat{q}=0\Rightarrow q=q_{d}$, so it was obtained thus:

(24) $$\begin{array}{cc} K_{I}\;\upsilon^{*}+K_{P}\;F_{p}\;\varphi_{p}^{*}+K_{D}\;F_{v}\;\varphi_{v}^{*} & =0 \end{array}$$

(25) $$\begin{array}{cc} \dot{\upsilon } & =F_{p}\;\varphi_{p}^{*}, \end{array}$$

where $\upsilon^{*}$, $\varphi_{p}^{*}$ and $\varphi_{v}^{*}$ were steady state values.

Subtracting (16) and (25) gave $\hat{q}=-F_{p}\left(\varphi_{p}-\varphi_{p}^{*}\right)$; then, subtracting (15) and (24), we obtained the error equations in the following form:

(26) $$M\left(q\right)\;\ddot{q}+C\left(q,\dot{q}\right)\;\dot{q}+K_{D}\;\dot{q}+K_{I}\;\hat{\upsilon }+K_{D}\;F_{v}\;{\hat{\varphi }}_{v}=0,$$

where $\hat{\upsilon }=\upsilon -\upsilon^{*}$ and ${\hat{\varphi }}_{v}=\varphi_{v}-\varphi_{v}^{*}$.

Following the methodology from [39,46], as the first step in the construction of the Lyapunov function, the error Equations (26) were multiplied on the left side by $\alpha {\hat{q}}^{T}+{\dot{q}}^{T}$ with some α>0:

(27) $$\begin{array}{cc} \; & \alpha {\hat{q}}^{T}\;M\left(q\right)\;\ddot{q}+\alpha {\hat{q}}^{T}\;C\left(q,\dot{q}\right)\;\dot{q}+\alpha {\hat{q}}^{T}\;K_{D}\;\dot{q}+\alpha {\hat{q}}^{T}\;K_{I}\;\hat{\upsilon }+\alpha {\hat{q}}^{T}\;K_{D}\;F_{v}\;{\hat{\varphi }}_{v}\\ \; & +{\dot{q}}^{T}\;M\left(q\right)\;\ddot{q}+{\dot{q}}^{T}\;C\left(q,\dot{q}\right)\;\dot{q}+{\dot{q}}^{T}\;K_{D}\;\dot{q}+{\dot{q}}^{T}\;K_{I}\;\hat{\upsilon }+{\dot{q}}^{T}\;K_{D}\;F_{v}\;{\hat{\varphi }}_{v}=0. \end{array}$$

Some of the terms in the expression (27) can be written as follows:

(28) $$\begin{array}{cc} {\dot{q}}^{T}\;M\left(q\right)\;\ddot{q} & =\frac{d}{dt}\left(\frac{1}{2}{\dot{q}}^{T}\;M\left(q\right)\;\dot{q}\right)-\frac{1}{2}{\dot{q}}^{T}\;\dot{M}\left(q\right)\;\dot{q}, \end{array}$$

(29) $$\begin{array}{cc} \alpha {\hat{q}}^{T}\;M\left(q\right)\;\ddot{q} & =\frac{d}{dt}\left(\alpha {\hat{q}}^{T}\;M\left(q\right)\;\dot{q}\right)-\alpha {\dot{q}}^{T}\;M\left(q\right)\;\dot{q}-\alpha {\hat{q}}^{T}\;\dot{M}\left(q\right)\;\dot{q}, \end{array}$$

(30) $$\begin{array}{cc} \alpha {\hat{q}}^{T}\;K_{D}\;\dot{q} & =\frac{d}{dt}\left(\frac{1}{2}\alpha {\hat{q}}^{T}\;K_{D}\;\hat{q}\right), \end{array}$$

(31) $$\begin{array}{cc} \alpha {\hat{q}}^{T}\;K_{I}\;\hat{\upsilon } & =\frac{d}{dt}\left(\frac{1}{2}\alpha {\hat{\upsilon }}^{T}\;K_{I}\;\hat{\upsilon }\right), \end{array}$$

(32) $$\begin{array}{cc} {\dot{q}}^{T}\;K_{I}\;\hat{\upsilon } & =\frac{d}{dt}\left({\hat{q}}^{T}\;K_{I}\;\hat{\upsilon }\right)-{\hat{q}}^{T}\;K_{I}\;\hat{q}. \end{array}$$

Inserting (28)–(32) in (27) and, as $\dot{M}\left(q\right)-2C\left(q,\dot{q}\right)$ was skew-symmetric (see Property 1), which implied ${\dot{q}}^{T}\left(\dot{M}\left(q\right)-2C\left(q,\dot{q}\right)\right)\dot{q}=0$, we obtained:

(33) $$\begin{array}{cc} \; & \frac{d}{dt}\left(\frac{1}{2}{\dot{q}}^{T}\;M\left(q\right)\;\dot{q}+\alpha {\hat{q}}^{T}\;M\left(q\right)\;\dot{q}+\frac{1}{2}\alpha {\hat{q}}^{T}\;K_{D}\;\hat{q}+\frac{1}{2}\alpha {\hat{\upsilon }}^{T}\;K_{I}\;\hat{\upsilon }+{\hat{q}}^{T}\;K_{I}\;\hat{\upsilon }\right)\\ \; & =\alpha {\dot{q}}^{T}\;M\left(q\right)\;\dot{q}+\alpha {\hat{q}}^{T}\;C{\left(q,\dot{q}\right)}^{T}\;\dot{q}-{\dot{q}}^{T}\;K_{D}\;\dot{q}+\alpha {\hat{q}}^{T}\;K_{I}\;\hat{q}-{\dot{q}}^{T}\;K_{D}\;F_{v}\;{\hat{\varphi }}_{v}-\alpha {\hat{q}}^{T}\;K_{D}\;F_{v}\;{\hat{\varphi }}_{v}. \end{array}$$

Based on the nonlinear differential form (33), it could be concluded that on the left-hand side we had the Lyapunov function candidate $V(\dot{q},\hat{q},\hat{\upsilon })$, while on the right-hand side we had a candidate for the time derivative of the Lyapunov function $\dot{V}\left(\dot{q},\hat{\upsilon },{\hat{\varphi }}_{v}\right)$.

The Lyapunov function candidate could be rewritten as follows:

(34) $$\begin{array}{cc} V(\dot{q},\hat{q},\hat{\upsilon }) & =\frac{1}{2}{\left(\alpha \hat{q}+\hat{\upsilon }\right)}^{T}\;M\left(q\right)\;\left(\alpha \hat{q}+\hat{\upsilon }\right)-\frac{1}{2}\alpha^{2}{\hat{q}}^{T}\;M\left(q\right)\;\hat{q}+\frac{1}{2}\alpha {\hat{q}}^{T}\;K_{D}\;\hat{q}\\ \; & +\frac{1}{2}{\left(\frac{1}{\sqrt{\alpha }}\hat{q}+\sqrt{\alpha }\hat{\upsilon }\right)}^{T}\;K_{I}\;\left(\frac{1}{\sqrt{\alpha }}\hat{q}+\sqrt{\alpha }\hat{\upsilon }\right)-\frac{1}{2\alpha }{\hat{q}}^{T}\;K_{I}\;\hat{q}. \end{array}$$

In order for the above expression to be positive-definite, it was necessary to determine the conditions under which

(35) $$-\frac{1}{2}\alpha^{2}{\hat{q}}^{T}\;M\left(q\right)\;\hat{q}+\frac{1}{2}\alpha {\hat{q}}^{T}\;K_{D}\;\hat{q}-\frac{1}{2\alpha }{\hat{q}}^{T}\;K_{I}\;\hat{q}>0.$$

Using definitions of vector and matrix norms, bound of quadratic forms (see Notation, Appendix A) and, finally, applying Property 2 we obtained the following:

(36) $$\left(\frac{1}{2}\alpha \lambda_{\min}\left\{K_{D}\right\}-\frac{1}{2\alpha }\lambda_{\max}\left\{K_{I}\right\}-\frac{1}{2}\alpha^{2}a_{1}\right){∥\hat{q}∥}^{2}>0,$$

which was positive-definite if

(37) $$\alpha \lambda_{\min}\left\{K_{D}\right\}-\frac{1}{\alpha }\lambda_{\max}\left\{K_{I}\right\}-\alpha^{2}a_{1}>0.$$

Next, according to the theory of $L_{2}$ stability and the passivity properties of Euler–Lagrange systems, the following inequality needed to be satisfied:

(38) $$\dot{V}\left(\dot{q},\hat{\upsilon },{\hat{\varphi }}_{v}\right)\leq \mu {∥\alpha \hat{q}+\dot{q}∥}^{2}-{\left(\alpha \hat{q}+\dot{q}\right)}^{T}\;{\hat{\varphi }}_{v},$$

where μ>0 was a finite $L_{2}$-gain.

Comparing (38) to the right-hand side of (33), using Cauchy–-Schwarz inequality, triangle inequality, definitions of vector and matrix norms, bound of quadratic forms (see Notation, Appendix A) and, finally, applying Properties 2 and 3, we got the following inequality:

(39) $$\begin{array}{cc} \; & \left(\alpha a_{1}+\left(c_{1}+d_{1}∥q∥\right)∥\hat{q}∥-\lambda_{\max}\left\{K_{D}\right\}-\mu \right)∥\dot{q}{∥}^{2}+\left(\alpha \lambda_{\min}\left\{K_{I}\right\}-\alpha \mu \right){∥\hat{q}∥}^{2}+\\ \; & \left(1-\lambda_{\max}\left\{K_{D}\right\}\sigma_{\max}\left\{F_{v}\right\}\right)∥\dot{q}∥\;∥{\hat{\varphi }}_{v}∥+\left(\alpha -\alpha \lambda_{\max}\left\{K_{D}\right\}\sigma_{\max}\left\{F_{v}\right\}\right)∥\hat{q}∥\;∥{\hat{\varphi }}_{v}∥\leq 0. \end{array}$$

The conditions that satisfied the above inequality were as follows:

(40) $$\begin{array}{c} \alpha a_{1}+\left(c_{1}+d_{1}∥q∥\right)∥\hat{q}∥-\lambda_{\max}\left\{K_{D}\right\}-\mu \leq 0, \end{array}$$

(41) $$\begin{array}{c} \alpha \lambda_{\min}\left\{K_{I}\right\}-\alpha \mu \leq 0, \end{array}$$

(42) $$\begin{array}{c} 1-\lambda_{\max}\left\{K_{D}\right\}\sigma_{\max}\left\{F_{v}\right\}\leq 0, \end{array}$$

(43) $$\begin{array}{c} \alpha -\alpha \lambda_{\max}\left\{K_{D}\right\}\sigma_{\max}\left\{F_{v}\right\}\leq 0. \end{array}$$

 □

**Remark** **4.** *As the condition (40) depended on the generalized coordinates of the system, it was concluded that the proposed controller guaranteed only local stability. For a more detailed analysis of stability, it would be necessary to determine the domain of attraction that guarantees asymptotic stability; furthermore, in order to ensure global stability, it would be necessary to introduce a nonlinear term in the control law (see, for example, [39]). Determining the domain of attraction and deriving the global stability conditions were beyond the scope of the research presented in this paper. The stability conditions derived in this work were calculated numerically, and served only for the appropriate initialization of the algorithm, thereby ensuring satisfactory convergence properties.*

## 5. Algorithm for the Controller Synthesis

In order to derive the algorithm for the controller synthesis, i.e., the algorithm to solve Problem 1, the PID control law (13) had to be written in parametrized form first. To do this, a vectorization operation was performed:

(44) $$\begin{array}{cc} \mathrm{vec}\left(u\right) & =-\mathrm{vec}\left(K_{P}\hat{q}\right)-\mathrm{vec}\left(K_{D}\dot{q}\right)-\mathrm{vec}\left(K_{I}\upsilon \right)-\mathrm{vec}\left(K_{P}F_{p}\varphi_{p}\right)-\mathrm{vec}\left(K_{D}F_{v}\;\varphi_{v}\right)\\ \; & =-\left({\hat{q}}^{T}⊗I\right)\mathrm{vec}\left(K_{P}\right)-\left({\dot{q}}^{T}⊗I\right)\mathrm{vec}\left(K_{D}\right)-\left(\upsilon^{T}⊗I\right)\mathrm{vec}\left(K_{I}\right)\\ \; & -\left(\varphi_{p}^{T}F_{p}^{T}⊗I\right)\mathrm{vec}\left(K_{P}\right)-\left(\varphi_{v}^{T}F_{v}^{T}⊗I\right)\mathrm{vec}\left(K_{D}\right)\\ \; & =-\left[\begin{array}{ccc} \left({\hat{q}}^{T}+\varphi_{p}^{T}F_{p}^{T}\right)⊗I & \left({\dot{q}}^{T}+\varphi_{v}^{T}F_{v}^{T}\right)⊗I & \upsilon^{T}⊗I \end{array}\right]\left[\begin{array}{c} \mathrm{vec}\left(K_{P}\right)\\ \mathrm{vec}\left(K_{D}\right)\\ \mathrm{vec}\left(K_{I}\right) \end{array}\right]. \end{array}$$

Then, by substituting $x={\left[\begin{array}{ccc} x_{1}^{T} & x_{2}^{T} & x_{3}^{T} \end{array}\right]}^{T}={\left[\begin{array}{ccc} {\hat{q}}^{T} & {\dot{q}}^{T} & \upsilon^{T} \end{array}\right]}^{T}$, from (15) and (16), we got the following first-order nonlinear differential equations:

(45) $$\dot{x}=f\left(x\right)+B\left(x,\varphi \right)k+F\varphi_{p},$$

where

(46) $$\begin{array}{cc} f(x) & =\left[\begin{array}{c} x_{2}\\ -M^{-1}\left(x_{1}\right)C\left(x_{1},x_{2}\right)x_{2}\\ x_{1} \end{array}\right],\\ B(x,\varphi_{p}) & =\left[\begin{array}{c} 0\\ -M^{-1}\left(x_{1}\right)R\left(x,\varphi \right)\\ 0 \end{array}\right],\\ R(x,\varphi_{p}) & =\left[\begin{array}{ccc} \left(x_{1}^{T}+\varphi_{p}^{T}F_{p}^{T}\right)⊗I & \left(x_{2}^{T}+\varphi_{v}^{T}F_{v}^{T}\right)⊗I & x_{3}^{T}⊗I \end{array}\right],\\ k & =\left[\begin{array}{c} \mathrm{vec}\left(K_{P}\right)\\ \mathrm{vec}\left(K_{D}\right)\\ \mathrm{vec}\left(K_{I}\right) \end{array}\right],\\ \varphi & =\left[\begin{array}{c} \varphi_{p}\\ \varphi_{v} \end{array}\right],\\ F & =\left[\begin{array}{c} 0\\ 0\\ F_{p} \end{array}\right]. \end{array}$$

Note that u=R(x,φ)k.

In accordance with the previous substitution, expression (17) then became

(47) $$J_{\mu }^{*}\left(x_{0}\right)=\min_{k}\max_{\varphi }\;\left\{∥x_{1}{∥}_{L_{2}}^{2}+{∥R\left(x,\varphi \right)k∥}_{L_{2}}^{2}-\mu {∥\varphi ∥}_{L_{2}}^{2}\right\},$$

where x, k and φ were coupled via nonlinear differential Equations (45) and (46).

### 5.1. $L_{2}$-Gain Minimization

The stability conditions from Proposition 1 suggested that a simple and well-known schematic bisection algorithm [55] could be applied, to minimize the $L_{2}$-gain: the steps of this method were as follows.

**Step initialization**: Choose the initial elements of the vector k, i.e., the initial PID controller gains, such that the lower $\mu_{l_{0}}$ and upper $\mu_{u_{0}}$ bound of the $L_{2}$-gain satisfy the conditions in expression (19). Choose a small enough positive constant, ϵ, as the stopping criteria of the bisection method.
**Step 1**: Set *k* := 1.
  **Step 2**: Set
  (48) $$\mu_{k}:=\frac{1}{2}\left(\mu_{l_{k-1}}+\mu_{u_{k-1}}\right),$$
  then calculate the value of function $J_{\mu_{k}}^{*}\left(x_{0}\right)$ by solving the zero-sum differential game (47).
**Step 3**: If $|J_{\mu_{k}}^{*}\left(x_{0}\right)|<\epsilon$, then stop; otherwise, if $J_{\mu_{k}}^{*}\left(x_{0}\right)\leq 0$ then $\mu_{l_{k}}:=\mu_{l_{k-1}}$ and $\mu_{u_{k}}:=\mu_{k}$; else, $\mu_{l_{k}}:=\mu_{k}$ and $\mu_{u_{k}}:=\mu_{u_{k-1}}$.
**Step 4**. Set k:=k+1 and return to **Step 2**.

### 5.2. Zero-Sum Differential Game Solution

In the second step of the algorithmic procedure described in the previous subsection, it was necessary to solve the problem defined by expression (47). To solve this zero-sum differential game, we implemented the Newton method, which is described in the following steps.

**Step initialization**: Choose a small enough positive constant, ε, as the stopping criterion of the Newton algorithm.
**Step 1**: Set j0:=.
**Step 2**: Determine the search direction vector $s_{j}$ by solving a system of linear equations using Cholesky factorization:
  (49) $$\left[\begin{array}{cc} \nabla_{k}^{2}J & \nabla_{k,\varphi }^{2}J\\ -\nabla_{\varphi ,k}^{2}J & -\nabla_{\varphi }^{2}J \end{array}\right]\;s_{j}=-\left[\begin{array}{c} \nabla_{k}^{T}J\\ -\nabla_{\varphi }^{T}J \end{array}\right],$$
  where *J* is the argument of the minmax operator in (47), $\nabla_{k}J$, $\nabla_{\varphi }J$, $\nabla_{k}^{2}J$, $\nabla_{\varphi }^{2}J$, and $\nabla_{k,\varphi }^{2}J$ are gradients and Hessians with respect to the vectors k and φ, respectively. Note that the maximization with respect to φ is achieved simply by the minus sign in front of the gradient and Hessians.
**Step 3**: Use the line search strategy satisfying the Wolfe conditions [56] to compute the step-size $\eta_{j}>0$.
**Step 4**: Calculate
  (50) $$\left[\begin{array}{c} k_{j+1}\\ \varphi_{j+1} \end{array}\right]=\left[\begin{array}{c} k_{j}\\ \varphi_{j} \end{array}\right]+\eta_{j}\;s_{j}.$$
**Step 5**: If
  (51) $${∥{\left[\begin{array}{cc} \nabla_{k}J & -\nabla_{\varphi }J \end{array}\right]}^{T}∥}_{\infty }\leq \varepsilon ,$$
  then stop; else, set j:=j+1 and go to **Step 2**.

As is well-known, Newton’s method has a locally quadratic convergence to the saddle point, assuming that the initial point is close enough to the saddle point. In the approach we propose here, the proximity to the saddle point is ensured by the $L_{2}$ stability conditions set in Proposition 1: this further implies that $\nabla_{k}^{2}J>0$ and $-\nabla_{\varphi }^{2}J>0$. As these Hessians are positive-definite submatrices on the main diagonal of the matrix on the left-hand side of (49), and submatrices $\nabla_{k,\varphi }^{2}J$ and $-\nabla_{\varphi ,k}^{2}J$ are negative transpositions of each other, the matrix on the left-hand side of Equation (49) is asymmetric positive-definite and, therefore, the linear system (49) is well-defined; therefore, the previously described algorithmic procedure, which is based on the Newton method, produces:

(52) $$\begin{array}{cc} k_{k} & \in \arg\;\min_{k}\left\{∥x_{1}{∥}_{L_{2}}^{2}+{∥R\left(x,\varphi \right)k∥}_{L_{2}}^{2}-\mu_{k}{∥\varphi ∥}_{L_{2}}^{2}\right\}, \end{array}$$

(53) $$\begin{array}{cc} \varphi_{k} & \in \arg\;\max_{\varphi }\left\{∥x_{1}{∥}_{L_{2}}^{2}+{∥R\left(x,\varphi \right)k∥}_{L_{2}}^{2}-\mu_{k}{∥\varphi ∥}_{L_{2}}^{2}\right\}, \end{array}$$

in the *k*-th iteration of the bisection algorithm proposed in Section 5.1.

To perform the steps of the proposed Newton-like algorithm, we needed expressions for the gradients and Hessians that appear in (49). A detailed procedure for deriving recursive matrix relations for computing these gradients and Hessians is given in the next subsection.

### 5.3. Matrix Relations for Recursive Calculation of Gradients and Hessians

In order to derive recursive relations for calculating gradients and Hessians, we used the fact that derivatives are a measure of the sensitivity of functions to small changes in their variables. This suggested that, for their calculation, we could perform a time discretization of the system dynamics (45). In the approach presented in this paper, the discretization of system dynamics using the fourth-order Adams approximation with a small time step was applied. In [32] it is shown that an explicit Adams method can be conveniently transformed into discrete-time state space.

The explicit Adams approximation of system (45) can be simply written in the following discrete-time state-space form:

(54) $$\hat{x}\left(i+1\right)=\hat{\phi }\left(\hat{x}\left(i\right),\;k\left(i\right),\varphi \left(i\right)\right),\;\;\hat{x}\left(0\right)={\hat{x}}_{0},$$

such that the time grid consists of points $t_{i}=t_{0}+i\tau$ for i=0,1,2,…,N−1, where $\tau =(t_{f}-t_{0})/N$ is the time step length, and $\hat{x}$ is the extended 4n-dimensional state vector (*n* is a dimension of state vector x in (45), and 4 is the order of the Adams method). More details on the Adams method can be found in [57].

The discrete-time form of the objective function resulted in

(55) $$J\left(x_{0}\right)=\tau \sum_{i=0}^{N-1}F\left(\hat{x}\left(i\right),k\left(i\right),\varphi \left(i\right)\right),$$

where *F* was the sub-integral function of the argument of the minmax operator in (47)

(56) $$F\left(i\right)=∥x_{1}{\left(i\right)∥}^{2}+{∥R\left(x\left(i\right),\varphi \left(i\right)\right)k\left(i\right)∥}^{2}-\mu {∥\varphi \left(i\right)∥}^{2}.$$

The gradient of the objective function (55), with respect to the k in the *j*-th iteration of the Newton algorithm (Section 5.2), was given by

(57) $$\nabla_{k(l)}J=\tau \sum_{i=0}^{N-1}\nabla_{k(l)}F\left(i\right)=\tau \left(\nabla_{k(l)}F\left(l\right)+\sum_{i=l+1}^{N-1}\nabla_{k(l)}F\left(i\right)\right),$$

for l=0,1,2,…,N−1. Note that, because of the causality principle, the terms for i<l were equal to zero.

As the terms under the sum on the right-hand side of (57) depended on k(l) implicitly through $\hat{x}\left(i\right)$ for i>l, this gave

(58) $$\nabla_{k(l)}F\left(i\right)=\nabla_{k(l)}\hat{x}\left(i\right)\;\nabla_{\hat{x}\left(i\right)}^{T}F\left(i\right).$$

Using the chain rule for ordered derivatives, from (54) it followed that

(59) $$\nabla_{k(l)}\hat{x}\left(i\right)=\nabla_{k(l)}\hat{x}\left(i-1\right)\;\nabla_{\hat{x}\left(i-1\right)}\hat{\phi }\left(i-1\right),$$

for i=l+2,…,N−1.

To simplify the following expressions, we introduced

(60) $$\sigma \left(l\right)=\sum_{i=l+1}^{N-1}\nabla_{k(l)}^{T}F\left(i\right).$$

Starting from l=N−2; i=N−1, it followed that

(61) $$\sigma \left(N-2\right)=\nabla_{k(N-2)}^{T}F\left(N-1\right),$$

then, substituting (58) in (61)

(62) $$\sigma \left(N-2\right)=\nabla_{k(N-2)}\hat{x}\left(N-1\right)\;\nabla_{\hat{x}\left(N-1\right)}^{T}F\left(N-1\right),$$

and substituting (59) in (62), and taking into account (54), we obtained

(63) $$\sigma \left(N-2\right)=\nabla_{k(N-2)}\hat{\phi }\left(N-2\right)\;\nabla_{\hat{x}\left(N-1\right)}^{T}F\left(N-1\right).$$

The above procedure could be further continued for l=N−3; i=N−1,N−2; l=N−4; i=N−3,N−2,N−1, etc., and the final recursive expressions for calculation of gradient $\nabla_{k}J$ had the following form:

(64) $$\begin{array}{cc} \omega (N-1) & =0, \end{array}$$

(65) $$\begin{array}{cc} \omega (l) & =\nabla_{\hat{x}\left(l+1\right)}^{T}F\left(l+1\right)+\nabla_{\hat{x}\left(l+1\right)}\hat{\phi }\left(l+1\right)\cdot \omega \left(l+1\right), \end{array}$$

(66) $$\begin{array}{cc} \sigma (l) & =\nabla_{k(l)}\hat{\phi }\left(l\right)\cdot \omega \left(l\right), \end{array}$$

(67) $$\begin{array}{cc} \nabla_{k(l)}J & =\tau \;\left(\nabla_{k(l)}F\left(l\right)+\sigma^{T}\left(l\right)\right), \end{array}$$

for l=N−2,N−3,…,0.

Before giving recursive expressions for computing the Hessians, we adopted the usual convention such that, for some function z=f(v), the Hessian is defined by $\nabla_{v}^{2}z=\nabla_{v}\left[\mathrm{vec}\left(\nabla_{v}z\right)\right]$. Then, in order to calculate $\nabla_{k}^{2}J$ in the *j*-th iteration of the Newton algorithm (Section 5.2), the derivatives of Equations (64)–(67), with respect to vectors $\hat{x}$ and k, were taken. We obtained the following recursive matrix relation:

(68) $$\begin{array}{cc} W(N-1) & =0, \end{array}$$

(69) $$\begin{array}{c} \begin{array}{cc} W(l) & =\nabla_{\hat{x}\left(l+1\right)}\left[\mathrm{vec}\left(\nabla_{\hat{x}\left(l+1\right)}^{T}F\left(l+1\right)\right)\right]\\ \; & +\nabla_{\hat{x}\left(l+1\right)}\left[\mathrm{vec}\left(\nabla_{\hat{x}\left(l+1\right)}\hat{\phi }\left(l+1\right)\right)\right]\cdot W\left(l+1\right), \end{array} \end{array}$$

(70) $$\begin{array}{cc} S(l) & =\nabla_{k(l)}\left[\mathrm{vec}\left(\nabla_{k(l)}\hat{\phi }\left(l\right)\right)\right]\cdot W\left(l\right), \end{array}$$

(71) $$\begin{array}{cc} \nabla_{k}^{2}\left(l\right)J & =\tau \;\left(\nabla_{k(l)}\left[\mathrm{vec}\left(\nabla_{k(l)}F\left(l\right)\right)\right]+S\left(l\right)\right), \end{array}$$

for l=N−2,N−3,…,0.

In the previous expressions, (64)–(71), the derivatives of the system dynamics (54) and sub-integral function (56), with respect to the vectors $\hat{x}$ and k,

(72) $$\begin{array}{cc} \; & \nabla_{\hat{x}\left(l+1\right)}\hat{\phi }\left(l+1\right),\;\nabla_{\hat{x}\left(l+1\right)}F\left(l+1\right),\;\nabla_{k(l)}\hat{\phi }\left(l\right),\;\nabla_{k(l)}F\left(l\right),\\ \; & \nabla_{\hat{x}\left(l+1\right)}\left[\mathrm{vec}\left(\nabla_{\hat{x}\left(l+1\right)}\hat{\phi }\left(l+1\right)\right)\right],\;\nabla_{\hat{x}\left(l+1\right)}\left[\mathrm{vec}\left(\nabla_{\hat{x}\left(l+1\right)}^{T}F\left(l+1\right)\right)\right],\\ \; & \nabla_{k(l)}\left[\mathrm{vec}\left(\nabla_{k(l)}\hat{\phi }\left(l\right)\right)\right],\;\nabla_{k(l)}\left[\mathrm{vec}\left(\nabla_{k(l)}F\left(l\right)\right)\right], \end{array}$$

were calculated by the hyper-dual number method [58,59], which enabled us to get the first- and second-order derivatives in one step, accurately to machine epsilon, which cannot be achieved by the finite difference method. Compared to the widely used and popular method of adjoint automatic differentiation, the hyper-dual number method is more robust and easier to apply.

In the previous considerations from expression (54)–(71) the derivation of recursive matrix relations for the calculation of gradients and Hessians, with respect to the vector k containing the gains of the PID controller, are shown. Matrix relations for calculating gradients and Hessians, with respect to vector φ containing the sensor faults, can be obtained by the same procedure, with obvious changes in notation, and there is no need to give them here.

**Figure 2:**
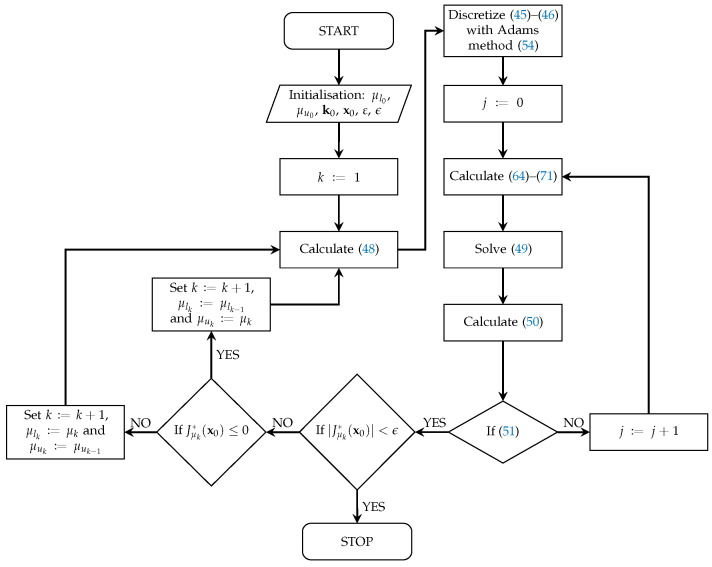
Flowchart of algorithmic procedure for calculation of PID controller gains and determination of the “worst case” of sensor fault functions.

A flowchart of the entire algorithmic procedure described in Section 5.1, Section 5.2 and Section 5.3 is shown in Figure 2.

## 6. Results and Discussions

### 6.1. Numerical Simulations

In this subsection, the simulation results for control of the robot manipulator, using the controller synthesis algorithm that is described in the previous sections, are presented.

**Figure 3:**
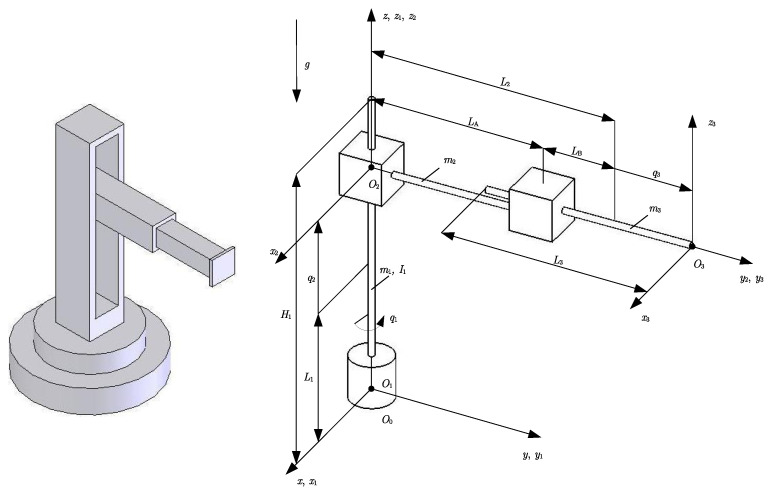
Computer model and schematic representation of a robot manipulator with three degrees of freedom.

Figure 3 shows the considered structure of a robot manipulator. The structure of a robot manipulator of this type was also the subject of consideration in [60,61]; however, for the sake of completeness, in this paper a more detailed derivation of the mathematical model is presented. Detailed expressions for kinetic and potential energy, and parameters that define the bounds of the robot’s dynamic properties, are given (see Appendix B and Appendix C).

The first joint is rotary, which enables the rotation of the robot around the vertical axis, while the second and third are prismatic joints, which enable movement in the vertical and horizontal directions, respectively. Such robot manipulators have a workspace in the shape of a cylinder. Robot manipulators with a cylindrical structure, due to their compact design, are most often used for simple assembly tasks, handling machine tools, applying coatings, etc. In addition, such robots can be fast, so a compromise should be sought, because speed implies problems with rotational inertia, which can affect repeatability if the entire system is not configured in accordance with its capabilities. It is usual for cylindrical robots that the up-and-down motion is achieved by pneumatic or hydraulic cylinders, while the rotation is usually achieved by electric motors and gears.

To model the kinematics and dynamics we denoted by $q_{1}$ (rad) the rotational coordinate of the first joint, and by $q_{2}$ (m) and $q_{3}$ (m) the translational coordinates of the second and third joints, respectively.

First, the forward kinematic equations for the cylindrical structure of a robot manipulator with 3DOF, using the Denavit–Hartenberg convention were developed. The Denavit–Hartenberg parameters are given in Table 1, where $\theta =q_{1}$, $d_{1}=0$, $d_{2}=L_{1}+q_{2}$ and $d_{3}=L_{2}+q_{3}$.

The homogeneous transformation matrices were as follows:

(73) $$\begin{array}{cc} A_{1} & =Rot\left(z,\;q_{1}\right)=\left[\begin{array}{cccc} \cos\left(q_{1}\right) & -\sin\left(q_{1}\right) & 0 & 0\\ \sin\left(q_{1}\right) & \cos\left(q_{1}\right) & 0 & 0\\ 0 & 0 & 1 & 0\\ 0 & 0 & 0 & 1 \end{array}\right], \end{array}$$

(74) $$\begin{array}{cc} A_{2} & =Tran\left(0,\;0,\;L_{1}+q_{2}\right)=\left[\begin{array}{cccc} 1 & 0 & 0 & 0\\ 0 & 1 & 0 & 0\\ 0 & 0 & 1 & L_{1}+q_{2}\\ 0 & 0 & 0 & 1 \end{array}\right], \end{array}$$

(75) $$\begin{array}{cc} A_{3} & =Tran\left(0,\;L_{2}+q_{3},\;0\right)=\left[\begin{array}{cccc} 1 & 0 & 0 & 0\\ 0 & 1 & 0 & L_{2}+q_{3}\\ 0 & 0 & 1 & 0\\ 0 & 0 & 0 & 1 \end{array}\right]. \end{array}$$

The transformation matrices were as follows:

(76) $$\begin{array}{cc} {}^{0}T_{1} & =A_{1}=\left[\begin{array}{cccc} \cos\left(q_{1}\right) & -\sin\left(q_{1}\right) & 0 & 0\\ \sin\left(q_{1}\right) & \cos\left(q_{1}\right) & 0 & 0\\ 0 & 0 & 1 & 0\\ 0 & 0 & 0 & 1 \end{array}\right], \end{array}$$

(77) $$\begin{array}{cc} {}^{0}T_{2} & =A_{1}A_{2}=\left[\begin{array}{cccc} \cos\left(q_{1}\right) & -\sin\left(q_{1}\right) & 0 & 0\\ \sin\left(q_{1}\right) & \cos\left(q_{1}\right) & 0 & 0\\ 0 & 0 & 1 & L_{1}+q_{2}\\ 0 & 0 & 0 & 1 \end{array}\right], \end{array}$$

(78) $$\begin{array}{cc} {}^{0}T_{3} & =A_{1}A_{2}A_{3}=\left[\begin{array}{cccc} \cos\left(q_{1}\right) & -\sin\left(q_{1}\right) & 0 & -\left(L_{2}+q_{3}\right)\sin\left(q_{1}\right)\\ \sin\left(q_{1}\right) & \cos\left(q_{1}\right) & 0 & \left(L_{2}+q_{3}\right)\cos\left(q_{1}\right)\\ 0 & 0 & 1 & L_{1}+q_{2}\\ 0 & 0 & 0 & 1 \end{array}\right]. \end{array}$$

where ${}^{0}T_{3}$ was the corresponding Denavit–Hartenberg matrix and, based on the last column, we had the position vector of the robot end-effector in the Cartesian space, as follows:

(79) $$\left[\begin{array}{c} p_{x}\\ p_{y}\\ p_{z} \end{array}\right]=\left[\begin{array}{c} -\left(L_{2}+q_{3}\right)\sin\left(q_{1}\right)\\ \left(L_{2}+q_{3}\right)\cos\left(q_{1}\right)\\ L_{1}+q_{2} \end{array}\right].$$

The solution of the inverse kinematic problem could be obtained, based on the following equation:

(80) $$\begin{array}{cc} A_{1}^{-1}\;{}^{0}T_{3} & =A_{2}A_{3}, \end{array}$$

(81) $$\begin{array}{cc} \left[\begin{array}{cccc} ⋆ & ⋆ & ⋆ & p_{x}\cos\left(q_{1}\right)+p_{y}\sin\left(q_{1}\right)\\ ⋆ & ⋆ & ⋆ & -p_{x}\sin\left(q_{1}\right)+p_{y}\cos\left(q_{1}\right)\\ ⋆ & ⋆ & ⋆ & p_{z}\\ 0 & 0 & 0 & 1 \end{array}\right] & =\left[\begin{array}{cccc} 1 & 0 & 0 & 0\\ 0 & 1 & 0 & L_{2}+q_{3}\\ 0 & 0 & 1 & L_{1}+q_{2}\\ 0 & 0 & 0 & 1 \end{array}\right]. \end{array}$$

Based on the last columns of the previous matrices on the left and right sides, we obtained:

(82) $$\begin{array}{cc} p_{x}\cos q_{1}+p_{y}\sin\left(q_{1}\right)=0⟹q_{1} & =\arctan\left(-\frac{p_{x}}{p_{y}}\right), \end{array}$$

(83) $$\begin{array}{cc} p_{z}=L_{1}+q_{2}⟹q_{2} & =-L_{1}+p_{z}, \end{array}$$

(84) $$\begin{array}{cc} -p_{x}\sin\left(q_{1}\right)+p_{y}\cos\left(q_{1}\right)=L_{2}+q_{3}⟹q_{3} & =-L_{2}-p_{x}\sin\left(q_{1}\right)+p_{y}\cos\left(q_{1}\right). \end{array}$$

In order to derive the corresponding dynamic equations of motion, the kinetic energy of the robot was first determined. The overall kinetic energy was calculated as the sum of kinetic energies from corresponding links, as follows:

(85) $$T=T_{1}+T_{2}+T_{3},$$

where

(86) $$\begin{array}{cc} T_{1} & =\frac{1}{2}I_{1}{\dot{q}}_{1}^{2}, \end{array}$$

(87) $$\begin{array}{cc} T_{2} & =\frac{1}{6}m_{2}\left(L_{A}^{2}{\dot{q}}_{1}^{2}+3{\dot{q}}_{2}^{2}\right), \end{array}$$

(88) $$\begin{array}{cc} T_{3} & =\frac{m_{3}}{2L_{3}}\left[\frac{1}{3}L_{3}^{3}{\dot{q}}_{1}^{2}+L_{3}^{2}\left(L_{2}+q_{3}\right){\dot{q}}_{1}^{2}+L_{3}\left({\left(L_{2}+q_{3}\right)}^{2}{\dot{q}}_{1}^{2}+{\dot{q}}_{2}^{2}+{\dot{q}}_{3}^{2}\right)\right]. \end{array}$$

Next, the overall potential energy was calculated, as the sum of potential energies from corresponding links, as follows:

(89) $$U=U_{1}+U_{2}+U_{3},$$

where

(90) $$\begin{array}{cc} U_{1} & =\frac{1}{2}m_{1}gH_{1}, \end{array}$$

(91) $$\begin{array}{cc} U_{2} & =m_{2}g\left(L_{1}+q_{2}\right), \end{array}$$

(92) $$\begin{array}{cc} U_{3} & =m_{3}g\left(L_{1}+q_{2}\right). \end{array}$$

The Euler–Lagrange equations of motion for a considered robot manipulator were given by

(93) $$\tau_{i}=\frac{d}{dt}\left(\frac{\partial T}{\partial {\dot{q}}_{i}}\right)-\frac{\partial T}{\partial q_{i}}+\frac{\partial U}{\partial q_{i}},\;\;i=1,2,3,$$

where *T* was the total kinetic energy defined by (85)–(88), *U* was the total potential energy defined by (89)–(92), and $\tau_{i}$ was the torque/force applied to the *i*-th robot link. Energy dissipation in rotational joints, and viscous friction in translational joints were neglected.

The Equation (93) can be written in matrix form (1), such that $q={\left[q_{1}\;\;q_{2}\;\;q_{3}\right]}^{T}$, $u={\left[\tau_{1}\;\;\tau_{2}\;\;\tau_{3}\right]}^{T}$ and

(94) $$\begin{array}{cc} M\left(q\right)\;\ddot{q} & =\left[\begin{array}{ccc} M_{11} & 0 & 0\\ 0 & m_{2}+m_{3} & 0\\ 0 & 0 & m_{3} \end{array}\right], \end{array}$$

(95) $$\begin{array}{cc} M_{11} & =I_{1}+L_{2}L_{3}m_{3}+L_{2}^{2}m_{3}+\frac{1}{3}\left(L_{A}^{2}m_{2}+L_{3}^{2}m_{3}\right)+\left(L_{3}m_{3}+2L_{2}m_{3}\right)q_{3}+m_{3}q_{3}^{2}, \end{array}$$

(96) $$\begin{array}{cc} C\left(q,\dot{q}\right)\;\dot{q} & =\left[\begin{array}{c} \left(L_{3}m_{3}+2L_{2}m_{3}+2m_{3}q_{3}\right){\dot{q}}_{1}\;{\dot{q}}_{3}\\ 0\\ \frac{1}{2}\left(-L_{3}m_{3}-2L_{2}m_{3}-2m_{3}q_{3}\right){\dot{q}}_{1}^{2} \end{array}\right], \end{array}$$

(97) $$\begin{array}{cc} g(q) & =\left[\begin{array}{c} 0\\ g\left(m_{2}+m_{3}\right)\\ 0 \end{array}\right]. \end{array}$$

A detailed derivation of expressions for the kinetic and potential energy of the considered robot manipulator is given in Appendix B.

The numerical values of the parameters relevant to the derivation of the dynamic model of the considered robot, using the Euler–Lagrange formalism, are given in Table 2.

Furthermore, for the appropriate initialization and numerical efficiency of the proposed algorithmic procedure for PID controller synthesis, we needed the constant values of the coefficients defined in properties (4)–(6). The numerical values of these parameters are given in Table 3, and the expressions for determining them for the considered cylindrical robot are given in Appendix C.

The parameters of the algorithm proposed in this paper, for this particular robot manipulator, were set as follows. The vector of initial conditions of the generalized coordinates was $q_{0}=0$. The initial values of the PID controller gaining satisfying stability conditions were chosen as

(98) $$K_{P0}=\left[\begin{array}{ccc} 120 & 0 & 0\\ 0 & 100 & 0\\ 0 & 0 & 50 \end{array}\right],\;K_{D0}=\left[\begin{array}{ccc} 110 & 0 & 0\\ 0 & 50 & 0\\ 0 & 0 & 30 \end{array}\right],\;K_{I0}=\left[\begin{array}{ccc} 50 & 0 & 0\\ 0 & 50 & 0\\ 0 & 0 & 20 \end{array}\right].$$

The stop criterion of the bisection method was chosen as $\epsilon =10^{-3}$. The criterion for stopping Newton’s method was set as $\varepsilon =10^{-4}$. The final time was $t_{f}=2$ s, and the number of optimization time intervals was N=2000, so that the sampling interval was τ=0.002 s.

The desired positions of the generalized coordinates were selected as follows: $q_{d1}=\pi /2$; $q_{d2}=0.2$ m; $q_{d3}=0.1$ m. By running the algorithm, we obtained the following PID controller gains:

(99) $$\begin{array}{cc} K_{P} & =\left[\begin{array}{ccc} 126.6367 & 0 & 0\\ 0 & 103.1782 & 0\\ 0 & 0 & 10.9068 \end{array}\right],\;K_{D}=\left[\begin{array}{ccc} 50.3292 & 0 & 0\\ 0 & 4.0766 & 0\\ 0 & 0 & 42.3659 \end{array}\right],\\ K_{I} & =\left[\begin{array}{ccc} 45.9922 & 0 & 0\\ 0 & 79.7230 & 0\\ 0 & 0 & 33.0817 \end{array}\right], \end{array}$$

and the minimum $L_{2}$-gain, μ=532.3096.

**Figure 4:**
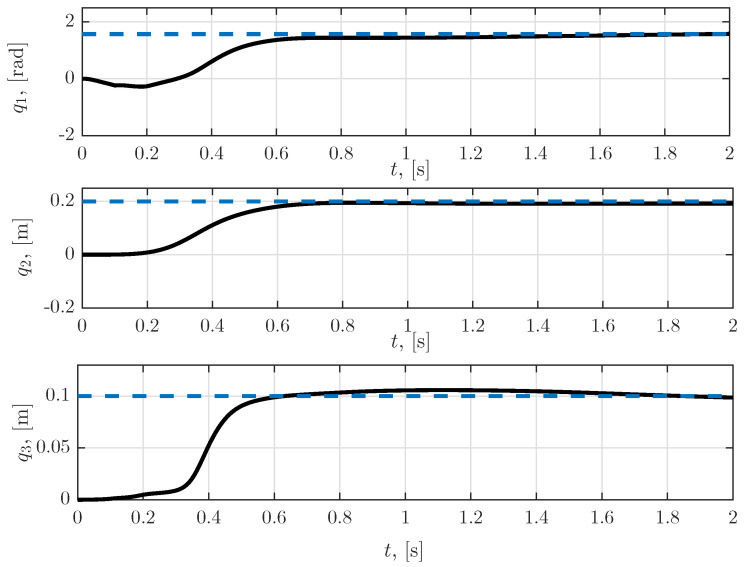
Time responses of the rotational coordinates of the first joint, and of the translational coordinates of the second and third joints.

The time responses of the rotational coordinates of the first joint $q_{1}$ and the translational coordinates of the second and third joints, $q_{2}$ and $q_{3}$, respectively, are presented in Figure 4. It can be seen from the figures that the transient response lasted approximately 0.8 s. The duration of the transient response could be further improved by adding weighting matrices into the objective function (47). When selecting these matrices, one should strive to reach a compromise between the time required to achieve the desired position, and the maximum value of the control torque/force, such that the effect of the sensor faults is kept under a permissible level, and ensures the stability of the closed-loop system.

**Figure 5:**
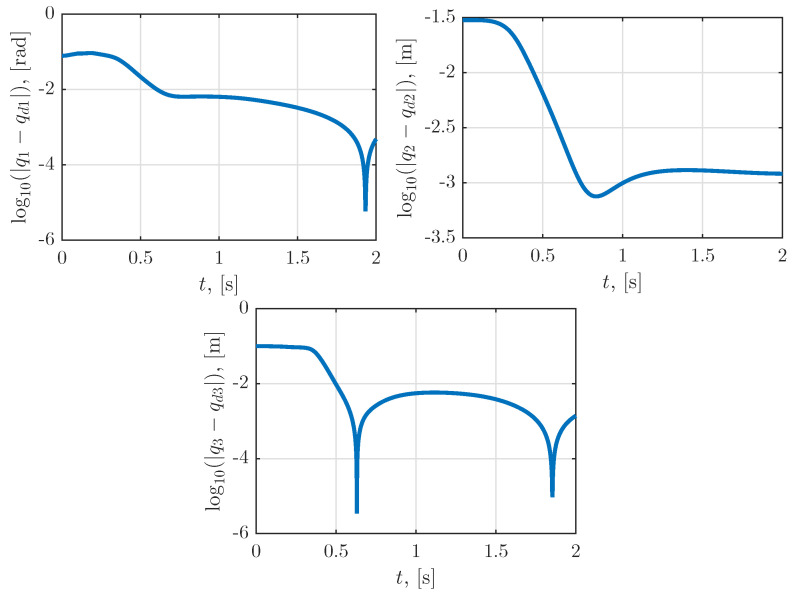
Time dependence of the positioning errors.

Figure 5 illustrates the time dependence of the positioning errors. It can be seen that the errors from the desired positions were approximately $10^{-3}$. These errors could be further reduced by reducing the parameters ϵ and ε of the bisection and Newton methods. Reducing these parameters would mean an increase in the number of iterations and, thus, the execution time of the algorithm. Furthermore, the positioning errors could also be reduced by choosing a smaller sampling time τ of the Adams method; however, as is well-known, there is a minimal value of τ that guarantees numerical stability.

**Figure 6:**
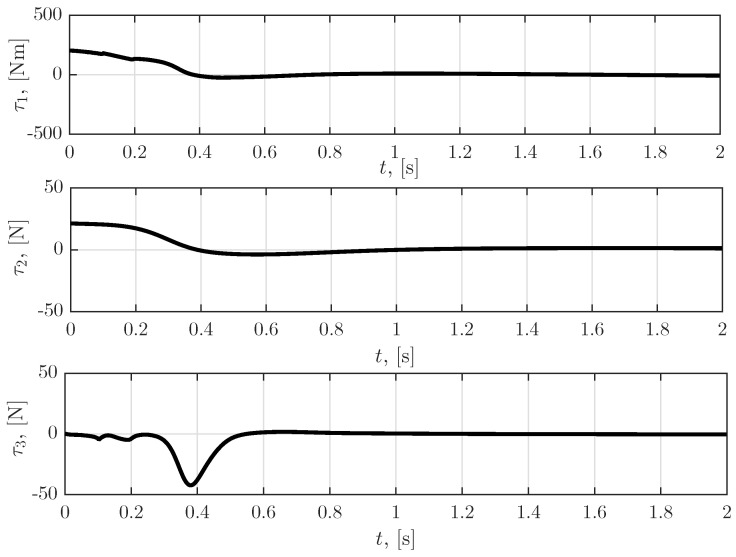
Time dependence of the force and torque applied to the robot links.

**Figure 7:**
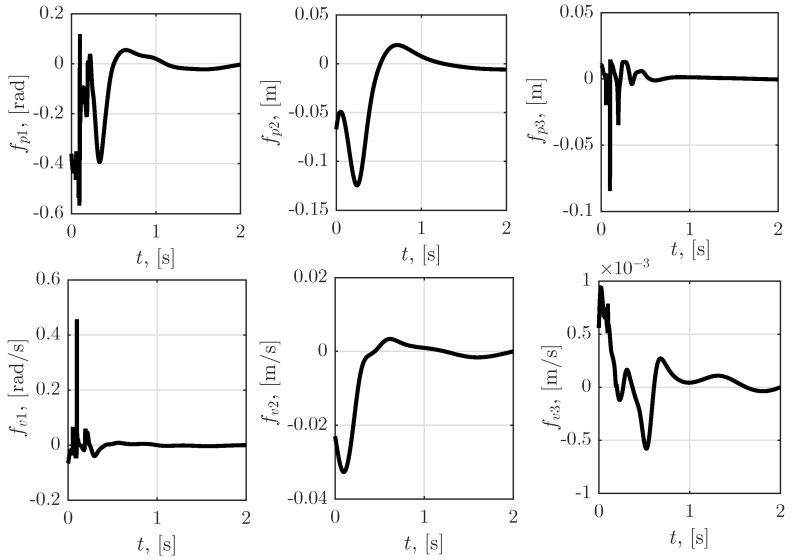
Time dependence of the “worst case” sensor faults obtained by proposed algorithms.

Figure 6 shows the time dependence of the force and torques applied to the robot links. Furthermore, Figure 7 illustrates the simulation results for the time dependence of the sensor faults calculated by the algorithm proposed in Section 5.1 and Section 5.2. Note that $f_{p1}$, $f_{p2}$, $f_{p3}$, $f_{v1}$, $f_{v2}$ and $f_{v3}$ are elements of vector φ, which is incorporated in the objective function of the zero-sum differential game (17), i.e., (47) and, since φ is the maximizing player, the sensor fault functions shown in Figure 7 affected the robot manipulator system in the worst possible manner. The obtained results can be interpreted as a sensor failure due to a deviation belonging to the class of bounded $L_{2}$ functions.

It is obvious from the figures that the robot manipulator reached the desired positions at an acceptable settling time, with a negligible steady state error and with acceptable amounts of applied forces and torques to achieve the desired motion: therefore, it can be concluded that the proposed control strategy is efficient in the presence of sensor faults.

### 6.2. Discussion of Comparison with Other Methods

Here, we discuss the features of our approach, in comparison to other similar approaches.

In fault-tolerant control approaches based on LMI formalism (see, for example, [6,7,23,29] and references therein), the nonlinear dynamics of the robot manipulator must be linearized, which means that robustness cannot be guaranteed in all operating points. Then, the decentralized PID controller must be transformed into a state feedback controller or an output feedback controller, and the optimization problem is presented in the form of an LMI, in which it is necessary to determine the elements of the positive-definite Lyapunov matrix and the transformation matrix. In the case of a robot with 3DOF, the Lyapunov matrix is 6×6 and the transformation matrix is 3×6: this means that the problem has at least 21+9 optimization parameters. In our approach, the PID controller gains were directly optimized, which meant that we only had 9 optimization parameters. Furthermore, the Mehrotra-type predictor–corrector variant of interior-point method algorithms is commonly used to solve the LMI problem: in our approach, we use Newton’s method, which—near the saddle point of the associated differential game—produces a sequence that quadratically converges to a desired solution, while the Mehrotra-type predictor–corrector has linear convergence. Thus, compared to methods based on solving LMI, the approach proposed in this paper has less optimization variables, and requires less iterations.

Many of the approaches to fault-tolerant control (see, for example, [2,6,12] and references therein), in addition to the synthesis of the controller, also imply a certain detection or estimation of the faults that appear in the nonlinear dynamic system: this actually means additional computational requirements that increase with the number of state variables that need to be estimated due to the synthesis of an additional subsystem. Compared to these approaches, our approach does not require an additional subsystem to determine how to modify the controller structure and gains, but focuses on the $L_{2}$-gain robust stability of the robot manipulator, considering the worst-case scenario of sensor faults rather than the desired performance for each fault occurrence scenario.

The algorithmic procedure presented in this paper is intended for the offline solution of the zero-sum differential game related to the $L_{2}$-gain optimal control problem, with the explicit calculation of the PID controller gains: therefore, the computational complexity of our approach is not as much of a limitation as for approaches intended for online execution, such as model predictive control (see, for example, [15,16] and references therein).

## 7. Conclusions

In this paper, the tuning of PID-type controller gains for robot manipulators affected by sensor faults, using an algorithmic procedure that gives an explicit solution to the $L_{2}$-gain optimality criterion and related zero-sum differential game, is presented. The main contribution of the paper includes the integration of the simple bisection method, the Newton method for solution of related zero-sum differential games without solving the HJI equation, the Adams method for time discretization, and hyper-dual numbers, to provide an effective method for control law synthesis. By applying Lyapunov stability analysis, and the dissipativity theory and passivity properties of systems described by Euler–Lagrange equations, we derived local $L_{2}$ stability conditions that we used for appropriate initialization of the bisection method, and for ensuring and controlling the convergence of Newton’s method. The simulation results for control of the 3DOF cylindrical robot manipulator showed that the proposed algorithmic procedure could efficiently calculate the controller gains, in order to position the system affected by sensor faults, as desired.

The extension of the proposed approach could be continued in the following directions:

- Although the case of sensor faults is considered in this paper, the proposed algorithm for control law synthesis could easily be extended to the case of dynamic systems affected by actuator faults, without significant increase in its complexity.
- Improvements in the control strategy proposed in this paper could also go towards the synthesis of a complete model-free control law, i.e., control law without gravity vector compensation: this would complicate the derivation of the stability conditions, and the controller gains should be presented as nonlinear functions of generalized coordinates.
- Instead of the initial conditions being known in advance, one could consider a case where the initial conditions were treated as an unknown uncertainty, i.e., the variables of the min–max optimization problem that maximizes the objective function.
- From the numerical optimization algorithms point of view, to perform a detailed analysis and comparison between a proposed algorithm and other existing methods, such as genetic algorithm or particle swarm optimization, in terms of convergence, accuracy and computational efficiency.
- From the point of view of its application to a robot manipulator affected by sensor faults, to carry out a detailed experimental analysis and comparison between a proposed control strategy and other existing related fault-tolerant control approaches.

## Figures and Tables

**Table 1:** Denavit–Hartenberg parameters of cylindrical manipulator.

**Parameter**	$\theta_{i}$	$d_{i}$	$a_{i}$	$\alpha_{i}$
**Joint 1**	$\theta_{1}$	$d_{1}$	0	0
**Joint 2**	0	$d_{2}$	0	π/2
**Joint 3**	0	$d_{3}$	0	0
**Unit**	rad	m	m	rad

**Table 2:** Numerical values of the robot’s constant parameters.

**Parameter**	$L_{2}$	$L_{3}$	$L_{A}$	$m_{2}$	$m_{3}$	$I_{1}$	*g*
**Value**	0.5	0.3	0.4	2.5	1	0.1	9.81
**Unit**	m	m	m	kg	kg	kgm${}^{2}$	m/s${}^{2}$

**Table 3:** Numerical values of the coefficients defined in properties (4)–(6).

**Parameter**	$a_{1}$	$a_{2}$	$c_{2}$	$d_{2}$	$c_{1}$	$d_{1}$
**Value**	0.2408	0.6633	1.3	1.0	0.9192	1.4142
**Unit**	kgm${}^{2}$	kgm${}^{2}$	kgm	kg	kgm	kg

## Data Availability

Not applicable.

## Abbreviations

The following abbreviations are used in this manuscript:

DOF	degrees of freedom
FTC	fault-tolerant control
HJI	Hamilton–Jacobi–Isaacs
LMI	linear matrix inequalities
PID	proportional–integral–derivative

## Appendix A. Notation

Matrices and vectors are represented in bold upper and bold lower case, respectively. Scalars are represented in italic lower case. I is an identity matrix, and 0 is a null matrix. The dimensions of the matrices and vectors can generally be determined trivially by the context. The symbols ∇ and $\nabla^{2}$ stand for the gradient and Hessian, respectively. The symbol ${}^{T}$ denotes transposition.

The vec(·) is an operator that stacks the columns of a matrix one underneath the other. The Kronecker product of the two matrices A(m×n) and B(p×q), denoted by A⊗B, is an mp×nq matrix defined by $A⊗B={\left(a_{ij}B\right)}_{ij}$. The definitions of the matrix differentials calculus, and the algebras related to Kronecker products, can be found in [62,63].

The Euclidean norm of vector is defined as $∥x∥=\sqrt{x^{T}\;x}$. $\lambda_{\min}\left\{A\right\}$ and $\lambda_{\max}\left\{A\right\}$ represent the smallest and the largest eigenvalues, respectively, of the symmetric positive-definite matrix A. The induced norm of matrix A is defined as $∥A∥=\sqrt{\lambda_{\max}\left\{A^{T}\;A\right\}}=\sigma_{\max}\left\{A\right\}$, where $\sigma_{\max}\left\{A\right\}$ represents the largest singular value of matrix A. If A is a symmetric positive-definite matrix, it implies $∥A∥=\lambda_{\max}\left\{A\right\}$; furthermore, for a symmetric positive-definite matrix, it holds that $\lambda_{\min}{\left\{A\right\}∥x∥}^{2}\leq x^{T}Ax\leq \lambda_{\max}\left\{A\right\}{∥x∥}^{2}$.

$L_{2}\left(I,R^{n}\right)$ stands for the standard Lebesgue spaces of vector-valued square-integrable and essentially bounded functions mapping an interval I⊂R to $R^{n}$: this space is equipped with an $L_{2}$ norm defined by ${∥\cdot ∥}_{L_{2}}=\sqrt{\int_{t_{0}}^{t_{f}}{∥\cdot ∥}^{2}dt}$. We avoided explicitly showing the dependence of the variables from the time when not needed.

## Appendix B. Detailed Derivation of Expressions for the Kinetic and Potential Energy of the Cylindrical Robot

The movement of the first link of the robot can be viewed as a rotation around an axis, so the kinetic energy can be determined as follows:

(A1) $$T_{1}=\frac{1}{2}I_{1}{\dot{q}}_{1}^{2},$$

where $I_{1}$ is dynamic moment of inertia $I_{1}=\frac{1}{2}m_{1}R^{2}$. The potential energy of the first link is defined by

(A2) $$U_{1}=-m_{1}g^{T}p_{1},$$

where $p_{1}={\left[\begin{array}{cccc} 0 & 0 & \frac{H_{1}}{2} & 1 \end{array}\right]}^{T}$ is the vector of the position of the center of gravity of the infinitesimal mass of the first link in relation to the fixed coordinate system, and $g={\left[\begin{array}{cccc} 0 & 0 & -g & 0 \end{array}\right]}^{T}$ is the vector of gravitational acceleration. By inserting them into the Equation (A2), we get

(A3) $$U_{1}=\frac{1}{2}gm_{1}H_{1}.$$

The kinetic energy of the second link is

(A4) $$T_{2}=\frac{1}{2}\int_{\left(m\right)}^{}v_{2}^{2}dm_{2}.$$

If we assume that the second link is homogeneous, then it follows:

(A5) $$\frac{dm_{2}}{m_{2}}=\frac{du_{2}}{L_{A}}\Rightarrow dm_{2}=\frac{m_{2}}{L_{A}}du_{2},$$

where $dm_{2}$ is infinitesimal mass and $du_{2}$ is infinitesimal displacement. Substituting expression (A5) into (A4) and changing the bounds of integration, it follows:

(A6) $$T_{2}=\frac{m_{2}}{2L_{A}}\int_{0}^{L_{A}}v_{2}^{2}du_{2}.$$

The position of the $dm_{2}$ in the robot coordinate system is determined by the position vector

(A7) $$R_{2}={\left[\begin{array}{cccc} 0 & u_{2} & 0 & 1 \end{array}\right]}^{T}.$$

The position vector of the mass $dm_{2}$, with respect to the fixed coordinate system, is

(A8) $$p_{2}={}^{0}T_{2}R_{2}.$$

By inserting expressions (77) and (A7) into expression (A8), we get

(A9) $$p_{2}={\left[\begin{array}{cccc} -u_{2}\sin\left(q_{1}\right) & u_{2}\cos\left(q_{1}\right) & L_{1}+q_{2} & 1 \end{array}\right]}^{T}.$$

The velocity of the mass $dm_{2}$ is

(A10) $$v_{2}=\frac{dp_{2}}{dt}={\left[\begin{array}{cccc} -u_{2}{\dot{q}}_{1}\cos\left(q_{1}\right) & -u_{2}{\dot{q}}_{1}\sin\left(q_{1}\right) & {\dot{q}}_{2} & 0 \end{array}\right]}^{T},$$

and the square of the velocity is

(A11) $$v_{2}^{2}=v_{2}\cdot v_{2}=u_{2}^{2}{\dot{q}}_{1}^{2}\cos^{2}\left(q_{1}\right)+u_{2}^{2}{\dot{q}}_{1}^{2}\sin^{2}\left(q_{1}\right)+{\dot{q}}_{2}^{2}.$$

Inserting Equation (A11) into (A6), and integrating from 0 to $L_{A}$, it follows:

(A12) $$\begin{array}{cc} T_{2} & =\frac{m_{2}}{2L_{A}}\left[\frac{L_{A}^{3}}{3}{\dot{q}}_{1}^{2}\cos^{2}\left(q_{1}\right)+\frac{L_{A}^{3}}{3}{\dot{q}}_{1}^{2}\sin^{2}\left(q_{1}\right)+L_{A}{\dot{q}}_{2}^{2}\right]\\ \; & =\frac{m_{2}}{2L_{A}}\left[\frac{L_{A}^{3}}{3}{\dot{q}}_{1}^{2}\left(\cos^{2}\left(q_{1}\right)+\sin^{2}\left(q_{1}\right)\right)+L_{A}{\dot{q}}_{2}^{2}\right]\\ \; & =\frac{1}{6}m_{2}\left(L_{A}^{2}{\dot{q}}_{1}^{2}+3{\dot{q}}_{2}^{2}\right). \end{array}$$

The potential energy of the second link is calculated as follows:

(A13) $$U_{2}=-m_{2}g^{T}p_{2},$$

where the vector $p_{2}$ is determined by (A9) for $u_{2}=L_{A}/2$, so the potential energy is equal to

(A14) $$U_{2}=-m_{2}\left[\begin{array}{cccc} 0 & 0 & -g & 0 \end{array}\right]\left[\begin{array}{c} -\frac{1}{2}L_{A}\sin\left(q_{1}\right)\\ \frac{1}{2}L_{A}\cos\left(q_{1}\right)\\ L_{1}+q_{2}\\ 1 \end{array}\right]=gm_{2}\left(L_{1}+q_{2}\right).$$

The same assumptions about the homogeneity of the second link and the linearity of the mass distribution are applied to the calculation of the kinetic and potential energy of the third link.

The kinetic energy of the third link is

(A15) $$T_{3}=\frac{1}{2}\int_{\left(m\right)}^{}v_{3}^{2}dm_{3},$$

and, with the $dm_{3}=\frac{m_{3}}{L_{3}}du_{3}$, it follows:

(A16) $$T_{3}=\frac{m_{3}}{2L_{3}}\int_{-L_{3}}^{0}v_{3}^{2}du_{3}.$$

The position vector of the infinitesimal mass $dm_{3}$ in the robot coordinate system is

(A17) $$R_{3}={\left[\begin{array}{cccc} 0 & -u_{3} & 0 & 1 \end{array}\right]}^{T}.$$

The position vector of the mass $dm_{3}$, with respect to the fixed coordinate system, is equal to

(A18) $$p_{3}={}^{0}T_{3}R_{3}=\left[\begin{array}{c} u_{3}\sin\left(q_{1}\right)-\left(L_{2}+q_{3}\right)\sin\;\left(q_{1}\right)\\ \left(L_{2}+q_{3}\right)\cos\left(q_{1}\right)-u_{3}\cos\left(q_{1}\right)\\ L_{1}+q_{2}\\ 1 \end{array}\right],$$

where the transfer matrix from the third coordinate system to the fixed coordinate system is defined by (78). The velocity of the mass $dm_{3}$ is

(A19) $$v_{3}=\frac{dp_{3}}{dt}=\left[\begin{array}{c} u_{3}{\dot{q}}_{1}\cos\left(q_{1}\right)-\left(L_{2}+q_{3}\right){\dot{q}}_{1}\cos\left(q_{1}\right)-{\dot{q}}_{3}\sin\left(q_{1}\right)\\ u_{3}{\dot{q}}_{1}\sin\left(q_{1}\right)-\left(L_{2}+q_{3}\right){\dot{q}}_{1}\sin\left(q_{1}\right)+{\dot{q}}_{3}\cos\left(q_{1}\right)\\ {\dot{q}}_{2}\\ 0 \end{array}\right],$$

while the square of the velocity is

(A20) $$v_{3}^{2}=v_{3}\cdot v_{3}={\dot{q}}_{1}^{2}{\left(L_{2}-u_{3}+q_{3}\right)}^{2}+{\dot{q}}_{2}^{2}+{\dot{q}}_{3}^{2}.$$

By inserting Equation (A20) into (A16), and after integrating within the limits from 0 to $-L_{3}$, we get kinetic energy of the third link as follows:

(A21) $$T_{3}=\frac{m_{3}}{2L_{3}}\left[\frac{1}{3}L_{3}^{3}{\dot{q}}_{1}^{2}+L_{3}^{2}\left(L_{2}+q_{3}\right){\dot{q}}_{1}^{2}+L_{3}\left({\left(L_{2}+q_{3}\right)}^{2}{\dot{q}}_{1}^{2}+{\dot{q}}_{2}^{2}+{\dot{q}}_{3}^{2}\right)\right].$$

The vector of the position of the center of gravity of the third link with respect to the fixed coordinate system is obtained by inserting $u_{3}=L_{3}/2$ into (A18):

(A22) $$p_{3}=\left[\begin{array}{c} \frac{1}{2}L_{3}\sin\left(q_{1}\right)-\left(L_{2}+q_{3}\right)\sin\left(q_{1}\right)\\ \left(L_{2}+q_{3}\right)\cos\left(q_{1}\right)-\frac{1}{2}L_{3}\cos\left(q_{1}\right)\\ L_{1}+q_{2}\\ 1 \end{array}\right]$$

The potential energy of the third link is calculated as follows:

(A23) $$U_{3}=-m_{3}g^{T}p_{3},$$

according to which, we get

(A24) $$\begin{array}{cc} U_{3} & =-m_{3}\left[\begin{array}{cccc} 0 & 0 & -g & 0 \end{array}\right]\left[\begin{array}{c} \frac{1}{2}L_{3}\sin\left(q_{1}\right)-\left(L_{2}+q_{3}\right)\sin\left(q_{1}\right)\\ \left(L_{2}+q_{3}\right)\cos\left(q_{1}\right)-\frac{1}{2}L_{3}\cos\left(q_{1}\right)\\ L_{1}+q_{2}\\ 1 \end{array}\right]\\ \; & =gm_{3}\left(L_{1}+q_{2}\right). \end{array}$$

## Appendix C. Properties of the Dynamic Model of the Cylindrical Robot

Based on the inertia matrix defined by (94) and (95), using expression (4) with $q={\left[q_{1}\;\;q_{2}\;\;q_{3}\right]}^{T}$ and $\xi ={\left[\xi_{1}\;\;\xi_{2}\;\;\xi_{3}\right]}^{T}$, the parameters $a_{1}$, $a_{2}$, $c_{2}$ and $d_{2}$ are determined as follows.

On the left-hand side of expression (4), we have

(A25) $$\begin{array}{cc} \; & \left[I_{1}+L_{2}L_{3}m_{3}+\frac{1}{3}\left(L_{A}^{2}m_{2}+L_{3}^{2}m_{3}\right)+L_{3}m_{3}q_{3}+m_{3}{\left(L_{2}+q_{3}\right)}^{2}\right]\xi_{1}^{2}\\ \; & +\left(m_{2}+m_{3}\right)\xi_{2}^{2}+m_{3}\xi_{3}^{2}\\ \; & \geq \left(-\frac{{\left(L_{3}m_{3}+2L_{2}m_{3}\right)}^{2}-4m_{3}p}{4m_{3}}\right)\xi_{1}^{2}+\left(m_{2}+m_{3}\right)\xi_{2}^{2}+m_{3}\xi_{3}^{2}\\ \; & \geq \min\left\{\left(-\frac{{\left(L_{3}m_{3}+2L_{2}m_{3}\right)}^{2}-4m_{3}p}{4m_{3}}\right),m_{2}+m_{3},m_{3}\right\}\left(\xi_{1}^{2}+\xi_{2}^{2}+\xi_{3}^{2}\right), \end{array}$$

where

(A26) $$p=I_{1}+L_{2}L_{3}m_{3}+L_{2}^{2}m_{3}+\frac{1}{3}\left(L_{A}^{2}m_{2}+L_{3}^{2}m_{3}\right).$$

from which it follows:

(A27) $$\begin{array}{cc} a_{1} & =\min\left\{\left(-\frac{{\left(L_{3}m_{3}+2L_{2}m_{3}\right)}^{2}-4m_{3}p}{4m_{3}}\right),m_{2}+m_{3},m_{3}\right\}\Rightarrow \\ a_{1} & =\min\left\{I_{1}+\frac{1}{12}L_{3}^{2}m_{3}+\frac{1}{3}L_{A}^{2}m_{2},m_{3}\right\} \end{array}$$

Furthermore, on the right-hand side of expression (4), we have

(A28) $$\begin{array}{cc} \; & \underset{p}{\underset{︸}{\left[I_{1}+L_{2}L_{3}m_{3}+L_{2}^{2}m_{3}+\frac{1}{3}\left(L_{A}^{2}m_{2}+L_{3}^{2}m_{3}\right)\right]}}\xi_{1}^{2}\\ \; & +\left[\left(L_{3}m_{3}+2L_{2}m_{3}\right)q_{3}+m_{3}q_{3}^{2}\right]\xi_{1}^{2}+\left(m_{2}+m_{3}\right)\xi_{2}^{2}+m_{3}\xi_{3}^{2}\\ \; & \leq \left[a_{2}+c_{2}\sqrt{q_{1}^{2}+q_{2}^{2}+q_{3}^{2}}+d_{2}\left(q_{1}^{2}+q_{2}^{2}+q_{3}^{2}\right)\right]\left(\xi_{1}^{2}+\xi_{2}^{2}+\xi_{3}^{2}\right). \end{array}$$

We can write the above expression as follows:

(A29) $$\begin{array}{cc} \; & \left[a_{2}-p+c_{2}\sqrt{q_{1}^{2}+q_{2}^{2}+q_{3}^{2}}-\left(L_{3}m_{3}+2L_{2}m_{3}\right)q_{3}+d_{2}\left(q_{1}^{2}+q_{2}^{2}+q_{3}^{2}\right)-m_{3}q_{3}^{2}\right]\xi_{1}^{2}\\ \; & +\left[a_{2}-\left(m_{2}+m_{3}\right)\right]\xi_{2}^{2}+\left[a_{2}-m_{3}\right]\xi_{3}^{2}\geq 0, \end{array}$$

from which it follows:

(A30) $$a_{2}\geq p,\;a_{2}\geq m_{2}+m_{3},\;a_{2}\geq m_{3},\;c_{2}\geq L_{3}m_{3}+2L_{2}m_{3},\;d_{2}\geq m_{3},$$

that is, we have

(A31) $$a_{2}=\min\left\{p,m_{3}\right\},\;c_{2}=L_{3}m_{3}+2L_{2}m_{3},\;d_{2}=m_{3}.$$

Based on the Coriolis vector (96), using expression (5), the parameters $c_{1}$ and $d_{1}$ are determined as follows.

First, we will square the expression (5):

(A32) $$∥C\left(q,\dot{q}\right)\dot{q}{∥}^{2}\leq (c_{1}+d_{1}{∥q∥)}^{2}{∥\dot{q}∥}^{4}.$$

On the left side of (A32) we have

(A33) $$∥C\left(q,\dot{q}\right)\dot{q}{∥}^{2}={\left[C\left(q,\dot{q}\right)\dot{q}\right]}^{T}C\left(q,\dot{q}\right)\dot{q}=h^{2}\left({\dot{q}}_{3}^{2}+\frac{1}{4}{\dot{q}}_{1}^{2}\right){\dot{q}}_{1}^{2},$$

where

(A34) $$h=L_{3}m_{3}+2L_{2}m_{3}+2m_{3}q_{3}.$$

Inserting (A33) into (A32) gives the following inequality:

(A35) $$h^{2}\left({\dot{q}}_{3}^{2}+\frac{1}{4}{\dot{q}}_{1}^{2}\right){\dot{q}}_{1}^{2}\leq b{\left({\dot{q}}_{1}^{2}+{\dot{q}}_{2}^{2}+{\dot{q}}_{3}^{2}\right)}^{2},$$

where

(A36) $$b={\left(c_{1}+d_{1}\sqrt{q_{1}^{2}+q_{2}^{2}+q_{3}^{2}}\right)}^{2}.$$

Furthermore, from (A35), it follows

(A37) $$\left(b-\frac{1}{4}h^{2}\right){\dot{q}}_{1}^{4}+2\left(b-\frac{1}{2}h^{2}\right){\dot{q}}_{1}^{2}{\dot{q}}_{3}^{2}+b{\left({\dot{q}}_{2}^{4}+{\dot{q}}_{3}^{4}\right)}^{2}+2b{\dot{q}}_{1}^{2}{\dot{q}}_{2}^{2}\geq 0,$$

which will be satisfied if

(A38) $$\begin{array}{cc} b & \geq \frac{1}{4}h^{2},\\ c_{1}+d_{1}\sqrt{q_{1}^{2}+q_{2}^{2}+q_{3}^{2}} & \geq \frac{\sqrt{2}}{2}\left[L_{3}m_{3}+2L_{2}m_{3}+2m_{3}q_{3}\right], \end{array}$$

so that, in the end, we get

(A39) $$c_{1}=\frac{\sqrt{2}}{2}L_{3}m_{3}+\sqrt{2}L_{2}m_{3},\;d_{1}=\sqrt{2}m_{3}.$$

The parameter $k_{g2}$ from (6) is equal to zero, because the considered cylindrical robot structure does not have translational degrees of freedom of motion at an angle that changes in relation to the gravitational field. As the gravitational vector defined by (97) is not a function of the controlled coordinates, it also follows from (6) that the parameter $k_{g1}$ is equal to zero.
